# Surface EMG-Based Inter-Session Gesture Recognition Enhanced by Deep Domain Adaptation

**DOI:** 10.3390/s17030458

**Published:** 2017-02-24

**Authors:** Yu Du, Wenguang Jin, Wentao Wei, Yu Hu, Weidong Geng

**Affiliations:** 1 State Key Lab of CAD&CG, College of Computer Science, Zhejiang University, Hangzhou 310027, China; answeror@zju.edu.cn (Y.D.); wentaowei@zju.edu.cn (W.W.); yudeshui@zju.edu.cn (Y.H.); 2 College of Information Science and Electronic Engineering, Zhejiang University, Hangzhou 310027, China; jinguang@zju.edu.cn

**Keywords:** muscle-computer interface, electromyography, gesture recognition, domain adaptation

## Abstract

High-density surface electromyography (HD-sEMG) is to record muscles’ electrical activity from a restricted area of the skin by using two dimensional arrays of closely spaced electrodes. This technique allows the analysis and modelling of sEMG signals in both the temporal and spatial domains, leading to new possibilities for studying next-generation muscle-computer interfaces (MCIs). sEMG-based gesture recognition has usually been investigated in an intra-session scenario, and the absence of a standard benchmark database limits the use of HD-sEMG in real-world MCI. To address these problems, we present a benchmark database of HD-sEMG recordings of hand gestures performed by 23 participants, based on an 8 × 16 electrode array, and propose a deep-learning-based domain adaptation framework to enhance sEMG-based inter-session gesture recognition. Experiments on NinaPro, CSL-HDEMG and our CapgMyo dataset validate that our approach outperforms state-of-the-arts methods on intra-session and effectively improved inter-session gesture recognition.

## 1. Introduction

A muscle-computer interface (MCI) [1] is an interaction methodology that directly transforms myoelectrical signals from mere reflections of muscle activities into interaction commands that convey the intent of the user’s movement. It does not rely on user actions performed on a physical device or on actions that are externally visible or audible, thus enabling an always-available input mechanism in myoelectric control.

Gesture recognition based on surface electromyography (sEMG) forms the technical core of non-intrusive MCIs. sEMG is a technique that measures a muscle’s electrical activity from the surface of the skin using one or more electrodes. Gesture recognition based on sEMG can be naturally framed as a pattern classification problem in which a classifier is usually trained through supervised learning. Existing gesture recognition approaches can be broadly divided into two categories: (1) methods based on sparse multi-channel sEMG and (2) methods based on high-density sEMG (HD-sEMG). Gesture recognition based on sparse multi-channel sEMG usually requires precise positioning of the electrodes over the muscle [2], thus limiting its use in MCIs. HD-sEMG (i.e., sEMG recorded using two-dimensional electrode arrays) has enabled both temporal and spatial changes of the electrical potential to be recorded by multiple, closely spaced electrodes on the skin overlaying a muscle area [3], and is relatively robust to positions of electrodes. In addition to uses in medical applications [4,5], HD-sEMG has also been used in recent years to recognize hand gestures for MCIs [2,6,7,8,9]. Despite the advances in HD-sEMG-based gesture recognition reported in the literature, its use remains limited primarily because of a lack of a standard benchmark database and because most studies use proprietary data, thus decreasing the reproducibility of the research results.

The problem becomes challenging in realistic situations in which the trained model is used to recognize gestures during a new recording session, because sEMG signals are highly subject specific and vary considerably even between recording sessions of the same user within the same experimental paradigm [10,11]. To ameliorate the effects of inter-session variability, data augmentation schemes [12,13] and model adaptation techniques [2,14,15,16] were developed. However, the problem of recognizing a large set of gestures in a new recording session is still far from being solved, both in terms of the recognition accuracy and the complexity of the calibration process.

HD-sEMG signals characterize the spatiotemporal distribution of myoelectric activity acquired by the electrode pickup placed over muscles. These signals can also provide a global view of the varying states of electric fields on the surface of the sampled muscles via arrays of electrodes over a muscle region. In other words, the instantaneous values of HD-sEMG present a relatively global measure of the physiological processes underlying muscle activities at a specific time. Our previous study [17] revealed that instantaneous electromyography data contain patterns that are reproducible across trials of the same gesture and that can serve to discriminate among different gestures for a group of individuals. Motivated by this, we converted the instantaneous values of HD-sEMG signals at each sampling instant to a grayscale image (in accordance with the electrode positioning), and thus, reframed the problem of recognizing hand gestures by instantaneous HD-sEMG images into a problem of image classification. Because the distributions of the sEMG signals vary considerably over different sessions [10,11], the recognition of hand gestures from instantaneous HD-sEMG images from different sessions can be accordingly formulated as a multi-source domain adaptation problem [18].

**Figure 1:**
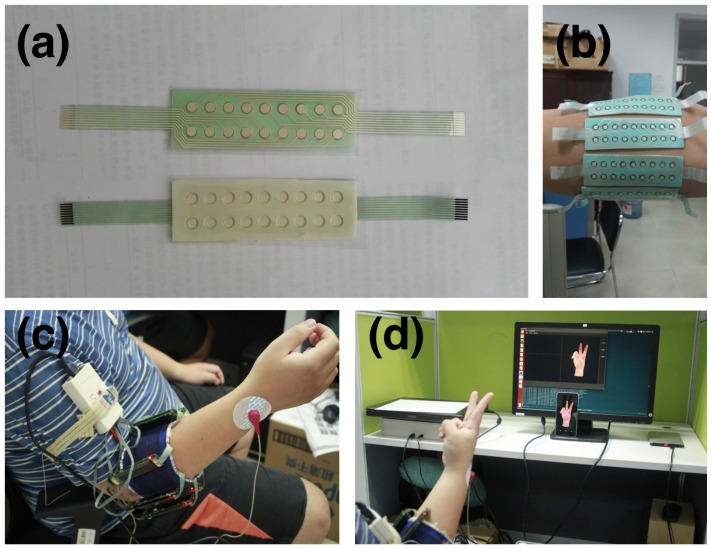
The acquisition setting-up: (**a**) The HD-sEMG electrode array; (**b**) 8 HD-sEMG electrode arrays on the right forearm; (**c**) The HD-sEMG acquisition device ready for capture; (**d**) The software subsystem to present the guided hand gesture and record HD-sEMG data simultaneously.

In this work, we describe the CapgMyo database, which includes HD-sEMG data for 128 channels acquired from 23 intact subjects by using our newly developed acquisition device [19]. The acquisition device, shown in Figure 1c, has a matrix-type (8 × 16) differential electrode array with silver wet electrodes (described in more detail in the acquisition setup section).

Our work consists of two major contributions.

1. We provide a new benchmark database for HD-sEMG-based gesture recognition. The CapgMyo database consists of 3 sub-databases (DB-a, DB-b and DB-c); 8 isometric and isotonic hand gestures were obtained from 18 of the 23 subjects in DB-a and from 10 of the 23 subjects in DB-b, and 12 basic movements of the fingers were obtained from 10 of the 23 subjects in DB-c.
2. We embedded a deep domain adaptation mechanism into the gesture classifier. When applied to new sessions/users, the adaptation starts working after the device is worn, and never stops until the user removes the device, going through the entire process of interaction (performing gesture recognition simultaneously).

Experiments on three benchmark datasets (NinaPro, CSL-HDEMG and our CapgMyo dataset) indicated that our approach outperforms other state-of-the-art methods with respect to both intra- and inter-session gesture recognition. To the best of our knowledge, our work is the first to address inter-session sEMG-based gesture recognition in an end-to-end framework based on instantaneous values of HD-sEMG signals.

### Related Work

The muscle-computer interface has received much attention from scientific communities and has been investigated using sEMG electrodes [1,2,20,21], pressure sensor [22,23], capacitive sensor [24,25], and ultrasound sensor [26]. The perceptual user interface based on sEMG is the predominant method in myoelectric control.

The recent emergence of high-density surface electromyography (HD-sEMG) [27,28,29], i.e., sEMG recorded with two-dimensional array systems, has enabled both temporal and spatial changes of the electrical potential to be recorded by several closely spaced electrodes on the skin overlying a muscle area [3]. HD-sEMG data consist of myoelectric signals that characterize the spatiotemporal distribution of myoelectric activity over the muscles that reside within the electrode pick-up area. HD-sEMG research originated in the 1970s. Lynn first extended the number of electrodes in a single direction to form a linear array to estimate the propagation velocity of the electrical activity along muscle fibres [30]. In the 1980s, two-dimensional arrays with a larger number of electrodes were developed for measuring muscle fibre conduction velocity [4] and for diagnostic purposes [5]. In addition to its use in medical applications, HD-sEMG has also been used in recent years to recognize hand gestures and for the proportional control of multiple degrees of freedom (DOFs) for muscle-computer interfaces (MCIs) [2,6,8,9,31,32,33].

Gesture recognition in sEMG-based MCIs can generally be divided into two categories based on the density of the employed electrodes. The first category is based on sparse multi-channel sEMG, which is typically posed as a sequence classification problem in which contiguous sequences of sEMG signals are assigned gesture labels. The classifier can be based on feature vectors extracted from a window of sEMG data [34,35,36], or based on temporal modeling (e.g., hidden Markov model) that analyzes sequential features from the sEMG data [37,38,39]. The involved features have been extensively evaluated, and accordingly, several feature sets have been proposed [40,41,42]. For conventional sparse multi-channel sEMG, there are several publicly available benchmark datasets [43,44,45,46,47]. Currently, the most widely accepted benchmark database is NinaPro [48], which was introduced in the development of hand prostheses and consists of a total of 52 gestures performed by 67 subjects–27 subjects in sub-database 1 (DB1) and 40 subjects in sub-database 2 (DB2)—recorded by using 10 sparsely located electrodes in DB1 and 12 sparsely located electrodes in DB2. The state-of-the-art recognition accuracy of 52 gestures in the NinaPro DB1 is 75.32% [48], which is insufficient for practical MCI systems.

The second category is based on high density surface EMG signals. Rojas et al. [6,7] used three electrode arrays (350 channels in total) at the upper arm and forearm to discriminate between 12 gesture classes corresponding to 4 task types and 3 effort levels. They defined an HD-sEMG map as a time-averaged 2D intensity map of HD-sEMG signals. Stango et al. [9] recognized hand and forearm movements by using an electrode array of 192 electrodes. This method achieved an accuracy of 95% for 9 classes; its classifier is based on spatial features of HD-sEMG maps, making it robust to electrode number and shift. With regard to HD-sEMG, the CSL-HDEMG benchmark database [2] was established specifically for sEMG-based gesture recognition. The sEMG signals in the CSL-HDEMG were recorded by using an electrode array with 192 electrodes, covering the upper forearm muscles of 5 subjects performing 27 gestures. However, the number of subjects in the CSL-HDEMG is relatively small – particularly in comparison with the NinaPro database—thus making it difficult to explore inter-subject variation and motivating us to develop a relatively large-scale HD-sEMG database. The recognition accuracies in this study reached 90.4% for intra-session evaluation and 58.9% for inter-session evaluation.

The aforementioned HD-sEMG-based gesture recognition methods required a handcrafted feature extractor to transform a window of sEMG signals into a feature vector or HD-sEMG map. Our previous study [17] has revealed that instantaneous electromyography data contain patterns that are reproducible across trials of the same gesture and that discriminate among different gestures for a group of individuals. In that study, we focused on per-frame gesture recognition and leverage a ConvNet with an architecture specifically designed to recognize hand gestures from instantaneous HD-sEMG images in an end-to-end way. We achieved state-of-the-art results on NinaPro, CSL-HDEMG and the first sub-database (DB-a) of our CapgMyo dataset. Atzori et al. [49] also employed a ConvNet-based gesture classifier to recognize hand gestures from a 150 ms window of sEMG signals in the NinaPro database. The recognition accuracy is lower than that of our per-frame approach (66.6% versus 76.1%).

From an MCI application scenario viewpoint, there are two types of gesture classifications: (1) intra-session, in which the classification model is trained on part of the data recorded from the subject during one session and evaluated on the another part of the data from that same session, and (2) inter-session, in which the classification model is trained on the data from all but a few sessions and evaluated on the remaining test set. Inter-session is often referred to as inter-subject when the training data and test data are from different subjects. The sEMG signals recorded in different sessions differ because of electrode shifts, changes in arm posture and slow time-dependent changes such as fatigue and electrode-skin contact impedance [10,11]. To reduce the effects of electrode shift, Hargrove et al. [12] augmented the training data by exploiting possible displacements. Amma et al. [2] estimated the electrode shift between sessions by using the Gaussian mixture model (GMM) and a small amount of calibration data for a specific gesture. Based on these techniques, the resulting inter-session recognition accuracy of 27 gestures was raised from 58.9% to 75.4%. Ju et al. [14] explored adaptive learning approaches with labeled calibration data. Khushaba [15] presented a canonical correlation analysis (CCA)-based algorithm for inter-subject gesture recognition. Patricia et al. [16] evaluated several adaptive learning algorithms using the NinaPro database and achieved an inter-subject recognition accuracy of approximately 40% for 52 gestures.

From a machine learning viewpoint, one of the key issues in inter-session MCIs is domain adaptation, i.e., developing learning algorithms in which the training data (source domain) used to learn a model have a different distribution compared with the data (target domain) to which the model is applied [18]. Domain adaptation has gained increasing interest in the context of deep learning [50,51,52,53,54,55]. When only a small amount of labeled data is available in the target domain during the training phase, fine-tuning pre-trained networks [50] has become the de facto method. For situations in which data in the target domain are not available or are unlabeled during the training phase, Long et al. [51] proposed a deep adaptation network (DAN) that is regularized by a multiple kernel variant of maximum mean discrepancies (MK-MMD). Tzeng et al. [52] employed an adversarial network [56] to simultaneously transfer task correlations and maximize domain confusion. Ganin et al. [53] simplified network training using a gradient reverse layer (GRL). Sun et al. [54] proposed transferring task correlations by aligning the second-order statistics in the deep features of the source domain with those in the target domain. Li et al. [55] further extended the method to each hidden layer by using AdaBN, which aligned the batch normalization [57] statistics between the source and target domains.

## 2. Materials and Methods

### 2.1. The CapgMyo Database

#### 2.1.1. Participants

We recruited 23 healthy, able-bodied subjects ranging in age from 23 to 26 years. Each subject was paid to perform a set of gestures with a non-invasive wearable acquisition device.

The study was conducted in accordance with the Declaration of Helsinki and was approved by the Ethics Committee of Zhejiang University, China. Written informed consent was obtained from all subjects.

#### 2.1.2. Acquisition Setup

We developed a non-invasive wearable device [19] to collect HD-sEMG data (shown in Figure 1c). This device consisted of 8 acquisition modules. Each acquisition module contained a matrix-type (8 × 2) differential electrode array (shown in Figure 1a), in which each electrode had a diameter of 3 mm and was arranged with an inter-electrode distance of 7.5 mm horizontally and 10.05 mm vertically. The silver wet electrodes were disposable and covered with conductive gel, with a contact impedance of less than 3 kΩ. The 8 acquisition modules were fixed around the right forearm with adhesive bands. The first acquisition module was placed on the extensor digitorum communis muscle at the height of the radio-humeral joint; others were equally spaced clockwise from the subject’s perspective, forming an 8 × 16 electrode array (shown in Figure 1b). The sEMG signals were band-pass filtered at 20–380 Hz and sampled at 1000 Hz, with a 16-bit A/C conversion. The resulting value was normalized to the [−1,1] range, corresponding to the voltage of [−2.5mV,2.5mV]. The sEMG data from the 8 acquisition modules were packed in an ARM controller and transferred to a PC via WIFI. The entire device was powered by a rechargeable lithium battery.

On the PC, our software displayed an animated 3D virtual hand [58] driven by pre-captured data from a data glove. The subjects were asked to mimic the hand gestures shown on the screen with their right hand (shown in Figure 1d); this software thus captured the sEMG data and labelled each frame in terms of the gesture performed by the virtual hand.

#### 2.1.3. Acquisition Protocol

Before the acquisition, subjects watched a tutorial video to familiarize themselves with the experiment. During the acquisition, subjects sat comfortably in an office chair and rested their hands on a desktop. Their skin was cleansed with rubbing alcohol prior to electrode placement, as recommended in previous studies [59,60,61]. The subjects were asked to mimic the gestures performed by the virtual hand shown on the screen by using their right hands (shown in Figure 1d). The interval between two consecutive recording sessions for the same subject, i.e., between doffing and donning the device, was at least one week.

As shown in Table 1, our set of gestures was a subset of the NinaPro database, with the same aim of incorporating the majority of the finger movements encountered in activities of daily living, which also made it possible to compare the performance of gesture recognition by using high density and sparse multi-channel sEMG signals. Each gesture was held for 3–10 s and repeated 10 times. To avoid fatigue, the gestures were alternated with a resting posture lasting 7 s. Because the gestures were performed in order, repetitive, almost unconscious movements were encouraged, as in the NinaPro database [48]. We didn’t enforce a pre-defined contraction force when a subject is performing gestures. From the point of view of gesture recognition, contraction force level is a kind of feature [40]. Enforcing the contraction force make it easier to recognize gestures from sEMG signals. Moreover, it’s hard to instruct users to have certain contraction force in real-world applications.

For each recording session, two additional max-force gestures were each performed once to estimate the maximal voluntary contraction (MVC) force level. In this study, we didn’t perform the registration with max-force data. We provided these two max-force gestures for the development of gesture recognition in inter-session and inter-subject scenarios in the future.

The CapgMyo database was divided into three sub-databases (denoted as DB-a, DB-b and DB-c) in terms of the acquisition procedure. DB-a contains 8 isometric and isotonic hand gestures obtained from 18 of the 23 subjects. The gestures in DB-a correspond to Nos. 13-20 in the NinaPro database. Each gesture in DB-a was held for 3 to 10 seconds. DB-b contains the same gesture set as in DB-a but was obtained from 10 of the 23 subjects. Every subject in DB-b contributed two recording sessions on different days, with an inter-recording interval greater than one week. As a result, the electrodes of the array were attached at slightly different positions each time. DB-c contains 12 basic movements of the fingers obtained from 10 of the 23 subjects. The gestures in DB-c correspond to Nos. 1–12 in the NinaPro database. Each gesture in DB-b and DB-c was held for approximately 3 s. To ensure lower skin impedance in DB-b and DB-c, the skin was abraded with soft sandpaper before being cleansed with alcohol.

Whereas DB-a was intended to fine-tune hyper-parameters of the recognition model, DB-b and DB-c were intended to evaluate intra-session and inter-session/inter-subject recognition algorithms. Inter-session recognition of hand gestures on the basis of sEMG typically suffers as a result of the shifting of the electrodes [2,10]. DB-b allows for the evaluation of methods to address this problem.

#### 2.1.4. Preprocessing

Power-line interference was removed from the sEMG signals by using a band-stop filter (45–55 Hz, second-order Butterworth) [62]. The label of each frame was assigned on the basis of the gesture performed by the guiding virtual hand in our acquisition software. Thus, the resulting gestures performed by the subjects may not perfectly match the label as a result of human reaction times. In this study, only the static part of the movement was used to evaluate the recognition algorithms. In other words, for each trial, the middle one-second window, i.e., 1000 frames of data, was used as described previously [63]. We use the middle one second data to ensure that no transition movements are included in training and testing. The raw data are also available in the online repository.

#### 2.1.5. Data Records

The data records are public available at http://zju-capg.org/myo/data. The format and content are described below.

The data records are in Matlab format. Each sub-database contains sss_ggg.mat for the raw data and sss_ggg_ttt.mat for the preprocessed data, where sss is the subject ID, ggg is the gesture ID, and ttt is the trial ID. For example, 004_001.mat contains the data (including the rest posture) from subject 4 performing gesture 1, and 004_001_003.mat contains the preprocessed 3rd trial.

The variables included in sss_ggg.mat and sss_ggg_ttt.mat are shown in Table 2. The IDs of the participants in each sub-database are shown in Table 3.

### 2.2. Inter-session Gesture Recognition by Deep Domain Adaptation

#### 2.2.1. Problem Statement

Let $S={\left\{\left(x_{i}^{s},y_{i}^{s}\right)\right\}}_{i=1}^{N_{s}}$, where $x^{s}\in R^{C}$ denotes the instantaneous HD-sEMG signals of the *C* channels in the training sessions and $y_{i}^{s}$ is the corresponding gesture label. Unlabeled data in the test sessions are denoted as $T={\left\{x_{i}^{t}\right\}}_{i=1}^{N_{t}}$. Gesture recognition with instantaneous HD-sEMG signals is a supervised classification problem in which a classifier $f_{\theta }$ is built to predict the hand gesture to which $x^{t}$ belongs, where *θ* is the unknown parameter estimated from S.

Let $A_{u}={\left\{x_{i}^{u}\right\}}_{i=1}^{N_{u}}\subset T$ denote the unlabeled calibration data. Gesture recognition with domain adaptation is an unsupervised classification problem in which a classifier $f_{\theta }$ is trained to predict the gesture label of $x^{t}$, where *θ* is first estimated from S and then adapted using $A_{u}$. When a small amount of labeled calibration data $A_{l}={\left\{x_{i}^{l},y_{i}^{l}\right\}}_{i=1}^{N_{l}}$ are available, where ${\left\{x_{i}^{l}\right\}}_{i=1}^{N_{l}}\subset T$ The trained classifier $f_{\theta }$ can be further adapted using $A_{l}$ by a supervised learning algorithm.

#### 2.2.2. Gesture Recognition by Deep Convolutional Network

In this section, we briefly describe the structure of the ConvNet-based classifier $f_{\theta }$ and the recognition scheme which were introduced in our previous work [17]. The values of HD-sEMG signals at each sampling instant were arranged in a two-dimensional grid in accordance with the electrode positioning. This grid was converted to a grayscale image using a linear transformation in which the units of the sEMG signal were converted from mV to a grayscale intensity. The details of the convertion for each database are described in Section 2.3.

**Figure 2:**
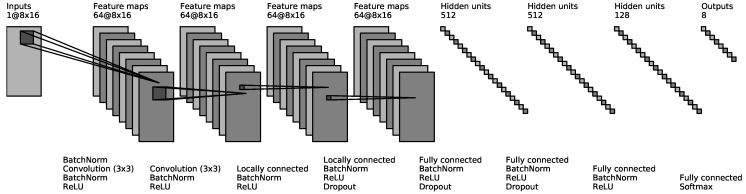
Outline of our ConvNet architecture. This is the ConvNet used for the recognition of 8 gestures from CapgMyo. The 8 × 16 input image is filtered by two convolution layers, followed by two locally connected layers and four fully connected layers. The boxes represent the inputs and outputs of different layers of the network. The text between the boxes describe the layers.

Our ConvNet had eight layers (shown in Figure 2). The input to the ConvNet consisted of a 7 × 24 image for CSL-HDEMG [2], a 8 × 16 image for CapgMyo and a 1 × 10 image for NinaPro [48]. The first two hidden layers were convolutional layers, each of which consisted of 64 3 × 3 filters with a stride of 1 and a padding of 1. The next two hidden layers were locally connected [64], each of which consisted of 64 non-overlapping 1 × 1 filters. The next three hidden layers were fully connected and consisted of 512, 512 and 128 units, respectively. The network ended with a *G*-way fully connected layer and a softmax function, where *G* is the number of gestures. We adopted (1) ReLU non-linearity [65] after each hidden layer, (2) batch normalization [57] after the input and before each ReLU non-linearity, and (3) dropout [66] with a probability of 0.5 after the fourth, fifth and sixth layers.

To prevent overfitting of small training set in some experiments, the ConvNet was initialized by pre-training using all available data when appropriate. We will discuss the details of pre-training in each experiment below.

In the recognition phase, the trained ConvNet was utilized to recognize hand gestures from HD-sEMG images frame by frame, minimizing the observational latency into 1 frame. Additionally, a majority voting scheme was used when two or more frames were available. Using this scheme, a window of sEMG signals was labeled with the class that received the most votes.

#### 2.2.3. Deep Domain Adaptation

Existing adaptive learning methods for sEMG-based gesture recognition faces the same challenge: devising an effective adaptation algorithm in high-dimensional non-linear space. Due to the capacity of the classification function and the computational constraints, most of the proposed methods are in the category of simple linear projections. Moreover, they often require users to undergo a rigorous calibration process.

**Figure 3:**
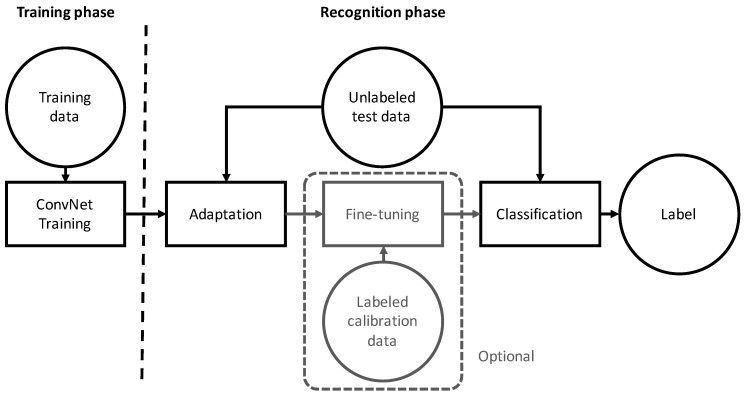
Gesture recognition based on deep domain adaptation. Fine-tuning is performed only when labeled calibration data are available.

The goal of domain adaptation is to develop a learning algorithm in which the training data in source domain S have a different distribution than the test data in target domain T. Motivated by the multi-source nature of the sEMG data (i.e., multiple training sessions or subjects), we formulated the recognition of hand gestures in instantaneous HD-sEMG images from different sessions as a multi-source domain adaptation problem, in which the classifier f(.) was learned from multiple domains in S, where each domain corresponded to one recording session. We propose two deep domain adaptation algorithms for inter-session gesture recognition, in which a classifier is trained using one of two algorithms or a combination of both, depending on whether the calibration data were labeled. A conceptual diagram of the proposed method is shown in Figure 3.

When the calibration data were unlabeled, we adopted AdaBN [55], which achieved state-of-the-art results using several benchmark datasets for image recognition. We first briefly review batch normalization [57] (BatchNorm), which is closely related to AdaBN. BatchNorm was originally developed to alleviate internal covariance shifting, which is a common problem when training a deep neural network. Internal covariance shifting refers to the phenomenon in which the distribution of the input to a layer changes as the parameters of the previous layers change during training. Internal covariance slows down the training and makes it difficult to train models with ReLU non-linearities. BatchNorm addresses this problem by normalizing the layer input with its mean and variance in each data batch. Given an input $U=\left[u_{1}\;u_{2}\cdots u_{n}\right]\in R^{m\times n}$, a BatchNorm layer transforms it into $V=\left[v_{1}\;v_{2}\cdots v_{n}\right]\in R^{m\times n}$, in which input feature *i* is transformed by

(1) $$v_{i}=\frac{\left(u_{i}-\mu_{i}\right)}{\sigma_{i}}\cdot \gamma_{i}+\beta_{i},$$

where *m* denotes the batch size, *n* is the feature dimension (n=1 when X is an input image), $\gamma_{i}$ and $\beta_{i}$ are parameters to be learned, and $\mu_{i}=E\left[X_{\cdot i}\right]$ and $\sigma_{i}^{2}=\mathrm{Var}\left[X_{\cdot i}\right]$ are the BatchNorm statistics for input feature *i*, where $X_{\cdot i}$ denotes the *i*-th column of X.

AdaBN was adopted under the hypothesis that the discriminating knowledge of different gestures is stored in the weights of each layer, whereas the discriminating knowledge of different recording sessions is represented by the statistics of the BatchNorm layer. The difference between AdaBN and fine-tuning [50] is that AdaBN don’t require the gesture labels of the adaptation data and AdaBN only update a small amount of network parameters (the statistics of BatchNorm) incrementally.

- In the training phase, the statistics $\mu_{i}$ and $\sigma_{i}$ for each source domain are calculated independently. Because the statistics were calculated for each data batch in the training phase, we only need to ensure that the samples in each data batch are from the same session.
- In the recognition phase, given the unlabeled calibration data $A_{u}$, AdaBN performs a forward pass, in which statistics $\mu_{i}$ and $\sigma_{i}$ of BatchNorm are updated with $E[X_{\cdot i}]$ and $\mathrm{Var}[X_{\cdot i}]$. The update is repeated for each BatchNorm, from bottom to top. If multiple batches of calibration data are presented, AdaBN performs multiple forward passes and calculate the statistics by moving average.

Li et al. [55] evaluated AdaBN with one or two source domains and achieved state-of-the-art results on benchmark datasets. However, we observed that the gradients fluctuate rapidly over data batches if many sessions were presented in the source domain S which may lead to lower recognition accuracy (verified in Section 3.3). For example, in inter-subject evaluation of CapgMyo DB-b, there are 9 recording sessions involved in the training phase.

Motivated by this, we propose a multi-stream extension to the classical AdaBN in the training phase. We evenly divided each data batch into *M* blocks and made sure that the samples in each block were from the same session. Let $X_{i:j}$ denote the submatrix of X from row *i* to row *j* and $X\left[k\right]=X_{(k-1)P+1:kP}$ denote the *k*-th row block of X, where P=m/M is the block size and k=1,2,⋯,M. We propose Algorithm 1 for AdaBN during the forward pass of each training iteration. The multi-stream AdaBN makes our network similar to multi-stream network, in which all streams share the same parameters except the statistics of BatchNorm. As a result, the number of statistics of multi-stream AdaBN is *M* times larger than that of classical AdaBN. To ensure that a data batch consists of data from various source domains, the blocks of training data were shuffled at each epoch.

In the recognition phase, the adaptation process is the same as classical AdaBN, i.e., the statistics of BatchNorm are updated with the unlabeled calibration data without block partition. Note, unlike fine-tuning, the statistics are updated incrementally by moving average and all other parameters of the network are fixed.

**Algorithm 1** Forward Pass of Multi-stream AdaBN in the traning phase **for**
i=1,2,⋯,n
**do**  **for**
k=1,2,⋯,M
**do**   $\mu_{i,k}\leftarrow E\left[U{\left[k\right]}_{\cdot i}\right]$   $\sigma_{i,k}^{2}\leftarrow \mathrm{Var}\left[U{\left[k\right]}_{\cdot i}\right]$   $V{\left[k\right]}_{i}\leftarrow \alpha_{i}\cdot \left(u{\left[k\right]}_{i}-\mu_{i,k}\right)/\sigma_{i,k}+\beta_{i}$  **end**
**for** **end**
**for**

In contrast to the aforementioned studies of MCIs, our adaptation method is unsupervised. Our method is in kind of incremental learning. When applied to new sessions or users, no explicit calibration of gestures is required, as the adaptation starts working after the device is worn, and never stops until the user unmounts the device, going through the entire process of interaction. The resulting accuracy is improved incrementally (shown in Section 3.2.2), well-fitted to inter-session sEMG gesture recognition. Our method does not require users to perform specific gestures during the calibration process. As the experiments in Section 3.2.2 shows, it only require a small and randomly selected subset of gestures to perform the adaptation.

When the calibration data were unlabeled, we first trained the ConvNet with multi-stream AdaBN and then fine-tuned it using the labeled calibration data $A_{l}$. Subsequently, unless explicitly stated, we used only the unlabeled calibration data and applied multi-stream AdaBN in the following experiments.

### 2.3. Experimental Setup

We evaluated our approach using three public databases: (1) the CSL-HDEMG database [2]; (2) the NinaPro database [48] (sub-database 1); and (3) our CapgMyo database. CSL-HDEMG [2] is for HD-sEMG-based gesture recognition. It contains HD-sEMG signals of 5 subjects performing 27 finger gestures, where each subject was recorded over 5 sessions and performed 10 trials for each gesture in each session. The sEMG signals were bipolar recorded at a sampling rate of 2048 Hz using an electrode array with 192 electrodes that covered the upper forearm muscles, forming a grid of 7 × 24 channels. The data were band-pass filtered and segmented as described in Amma et al. [2]. In our evaluation, the sEMG signals of CSL-HDEMG at each sampling instant were preprocessed using a 3 × 3 spatial median filter. For our CapgMyo database and the CSL-HDEMG database, an instantaneous sEMG image was formed as a grayscale image (8 × 16 for CapgMyo and 7 × 24 for CSL-HDEMG) by linearly transforming the values of sEMG signals from [0,1] to [0,255].

The NinaPro sub-database 1 (DB-1) [48] is for the development of hand prostheses, and contains sparse multi-channel sEMG recordings. It consists of a total of 52 gestures performed by 27 intact subjects, recorded at a sampling rate of 100 Hz, using 10 sparsely located electrodes placed on subjects’ upper forearms. The sEMG signals were filtered and smoothed by the acquisition device. We transformed the values of the sEMG signals at each instant into an image with 1 × 10 pixels, where the first eight components corresponded to the equally spaced electrodes around the forearm at the height of the radiohumeral joint and each of the last two components corresponded to electrodes placed on the main activity spots of the flexor digitorum superficialis and the extensor digitorum superficialis.

The deep-learning framework is based on MxNet [67], a multi-language machine learning library intended to simplify the development of ML algorithms, especially for deep-learning. We used stochastic gradient descent (SGD) [68] with a batch size of 1000, an epoch number of 28, and a weight decay of 0.0001 in all the experiments. The number of streams of multi-stream AdaBN (*M*) was set to 10. The learning rate started at 0.1 and was divided by 10 after the 16th and 24th epochs. The weights of the ConvNet were initialized as described in [69] when a pre-trained ConvNet was not available. For all experiments that involves a majority voting window, the sliding window moves forward one frame in each step.

**Figure 4:**
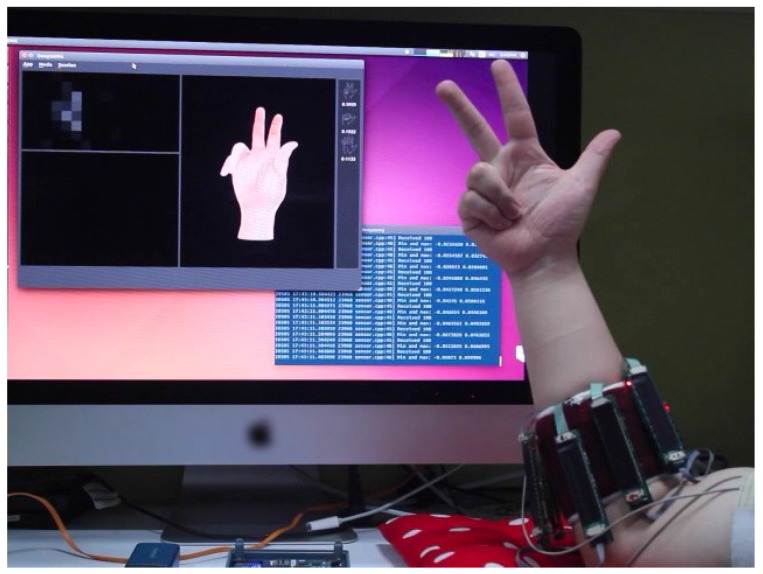
The HD-sEMG-based gesture recognition system. The software subsystem displays the recognized hand gesture and the recorded HD-sEMG image in real-time.

We also developed a real-time gesture recognition system, shown in Figure 4, which recognizes 8 isometric and isotonic finger gestures (equivalent to Nos. 13–20 in NinaPro [48]) using the 128 channels HD-sEMG signals recorded by our non-invasive wearable device. Our gesture recognition software displays the recognized hand gesture and the recorded HD-sEMG image in real-time. It took approximately 0.5 ms to process one frame of HD-sEMG signals on a workstation with one NVidia Titan X GPU.

## 3. Results

Our evaluation consists of two parts. The first part is the verification of the technical quality of our CapgMyo database. In this part, we compared the recognition accuracy with that of the NinaPro database, using the same classification scheme, to show that state-of-the-art classifiers can leverage HD-sEMG and obtain higher recognition accuracies, demonstrating the superiority of HD-sEMG. The second part is the inter-session evaluation on three databases. The second part is motivated by the huge difference of accuracy between intra-session and inter-session gesture recognition on CSL-HDEMG [2] and CapgMyo (shown in Table 4). We focus on how inter-session recognition is enhanced by our deep-learning-based domain adaptation framework and briefly describe the evaluation protocol and results of intra-session recognition for future comparisons. We refer the reader to [17] for more details for intra-session evaluation on CapgMyo DB-a, CSL-HDEMG [2] and NinaPro [48]. We demonstrated the advantages of the proposed adaptation scheme compared to state-of-the-art methods [2,16] in terms of both the gesture recognition accuracy and the complexity of the calibration process.

### 3.1. Technical Validation of CapgMyo

To verify that the data in CapgMyo allow the recognition of finger gestures, we applied our ConvNet-based classifier and four conventional classification methods to the instantaneous values of HD-sEMG signals in a intra-session setting. Moreover, we compared the recognition accuracy with that of the NinaPro database, using the same classification scheme, to show that state-of-the-art classifiers can leverage HD-sEMG and obtain higher recognition accuracies, demonstrating the superiority of HD-sEMG.

The evaluation procedure followed that of our previous study [17], but the training set was not downsampled. Therefore, we re-evaluated DB-a in this study. For each subject, a classifier was trained by using 50% of the data (e.g., trials 1, 3, 5, 7 and 9 for that subject) and tested by using the remaining half. This procedure was performed on each sub-database. For DB-b, the second session of each subject was used for the evaluation. We evaluated the proposed ConvNet and four conventional classifiers: KNN (k-nearest neighbours), SVM (support vector machine), Random Forest and LDA (linear discriminant analysis). For conventional classifiers, we used the implementation and default hyper-parameters of Scikit-learn 0.17.0 [70].

**Figure 5:**
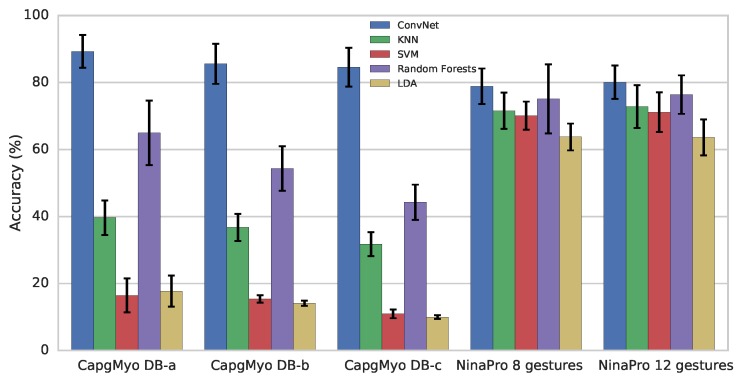
Recognition accuracy of hand gestures with instantaneous values of sEMG signals for different datasets and recognition approaches. Each group of columns represents a specific experiment. Different colours represent different recognition approaches: ConvNet (deep convolutional network) with instantaneous sEMG images, KNN (k-nearest neighbours), SVM (support vector machine), Random Forests and LDA (linear discriminant analysis). Bars denote average recognition accuracies over different subjects. Error bars denote standard deviations.

As shown in Figure 5, using DB-a and DB-b for recognition of 8 gestures resulted in similar accuracy. The highest recognition accuracies were 89.3% for DB-a and 85.6% for DB-b and were obtained with ConvNet. The highest recognition accuracy for the 12 gestures of DB-c was 84.6%, obtained with ConvNet. The performance difference of the conventional classifiers over NinaPro and CapgMyo are due to two reasons: (1) The number of sEMG channels in CapgMyo is much greater than that in NinaPro (128 versus 10). The ConvNet was carefully designed for HD-sEMG. Conventional classifiers (with default hyper-parameters) may not work well on the raw HD-sEMG signals directly. For conventional classifiers, we used the implementation and default hyper-parameters of Scikit-learn 0.17.0 [70], as that in our previous study [17]. The SVM performed not well partly because the default configuration has a linear kernel (LinearSVC in Scikit-learn). (2) The sEMG signals of NinaPro DB1 have been rectified and smoothed by the acquisition device. The signals of CapgMyo are neither rectified nor smoothed. The signals of CapgMyo are noisier than that of NinaPro, and thus require more complex model to recognize gestures from them.

**Figure 6:**
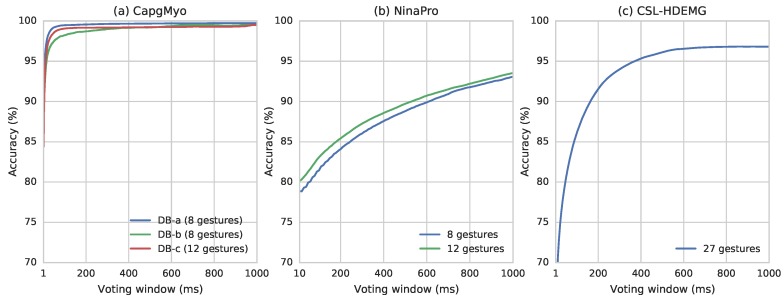
Surface EMG gesture recognition accuracy with different voting windows using ConvNet. (**a**) Recognition accuracy of gestures in CapgMyo; (**b**) Recognition accuracy of two subsets of gestures in NinaPro DB1; (**c**) Recognition accuracy of 27 gestures in CSL-HDEMG (Reprinted from [17]).

Based on simple majority voting algorithm, we have observed very good recognition rate (Figure 6). The recognition accuracy reached 90.0% for all sub-databases with no more than 5 frames, and reached 95.0% by simple majority voting over 7 frames for DB-a, 16 frames for DB-b and 12 frames for DB-c. Higher recognition accuracies of 99.5%, 98.6% and 99.2% can be obtained by simple majority voting over the recognition result of 150 frames for DB-a, DB-b and DB-c, respectively. At our sampling rate, 150 frames is equivalent to 150 ms, which is the window size suggested by several studies of pattern recognition based on prosthetic control [11,71,72]. The recognition accuracy of 27 gestures in CSL-HDEMG reached 55.8% on a single frame of HD-sEMG signals, and it reached 89.3%, 90.4% and 95.0% using simple majority voting over 307, 350 and 758 frames, respectively, with a 2048 Hz sampling rate [2].

### 3.2. Evaluation using CSL-HDEMG

#### 3.2.1. Intra-session evaluation

We have briefly reported the average intra-session recognition accuracy on the CSL-HDEMG database in our previous study [17]. Here we present the details of the pre-training and recognition accuracy for each subject in each recording session. We used the same evaluation procedure as was used in the previous study [2]. For each recording session, we performed a leave-one-out cross-validation, in which each of the 10 trials was used in turn as the test set and a classifier was trained by using the remaining 9 trials. The ConvNet was pre-trained with all available data for the target subject. For example, in the 1st round of the cross-validation of the 2nd subject’s 3rd session, the ConvNet was pre-trained using the 2nd to 10th trials of all sessions of the 2nd subject and tested using the 1st trial of the 2nd subject’s 3rd session. As shown in Table 5, our method achieved an accuracy of 96.8%, an 6.4% improvement over the latest work [2].

#### 3.2.2. Inter-session evaluation

We used the same evaluation procedure as in the previous study [2]. For each subject, we performed a leave-one-out cross-validation, in which each of the 5 sessions was used in turn as the test set and a classifier was trained using the remaining 4 sessions. The ConvNet was pre-trained using all the available data from all subjects. For example, in the 1st round of the cross-validation of the 2nd subject, the ConvNet was pre-trained using the 2nd to 5th sessions of all subjects and tested using the 1st session of the 2nd subject. Our adaptation scheme enhanced inter-session recognition with an 19.6% improvement (shown in Table 4). As shown in Table 6, our method achieved an accuracy of 82.3%, an 6.9% improvement over the latest work [2] with calibration.

The recognition accuracy reached 35.4% for a single frame of HD-sEMG signals, and it reached 58.9%, 69.3% and 75.4% with simple majority voting over 98, 307 and 539 frames, respectively, with a 2048 Hz sampling rate.

We also evaluated a data augmentation scheme that randomly shifted the training images by one pixel in four directions and filled the borders with the marginal values. This augmentation raised the recognition accuracies using per-trial voting from 62.7% to 68.3% without adaptation, and raised it from 82.3% to 85.0% with adaptation. Unless explicitly mentioned, we did not augment the training set in the following experiments.

**Figure 7:**
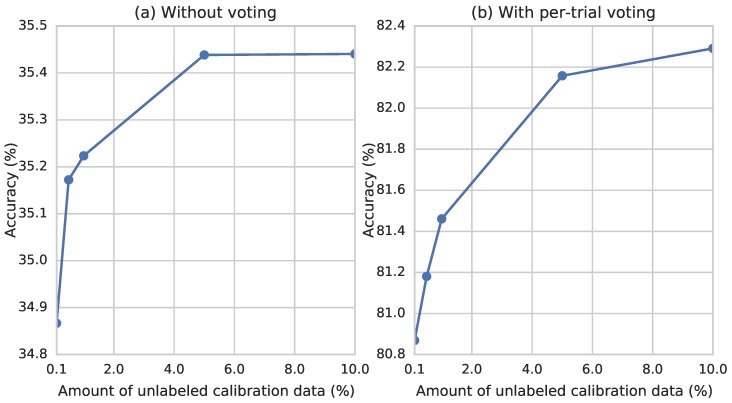
Recognition accuracies with different amounts of unlabeled calibration data for inter-session evaluation of CSL-HDEMG.

In the above experiments, we used the entire unlabeled test set as the calibration data for adaptation. It is natural to ask how much data is required to obtain a stable recognition accuracy. Therefore, we randomly selected a subset (0.1%, 0.5%, 1%, 5% and 10%) of the unlabeled test set to perform domain adaptation and then evaluate the performance. As shown in Figure 7, the recognition accuracy peaks after observing approximately 5% of the calibration data, which comprises approximately 20,000 frames–approximately 10 seconds at a sampling rate of 2,048. This suggests that our gesture recognition model could quickly be fitted to a user in a new recording session.

Our method also works if some categories weren’t performed yet during the process of interaction. The per-frame inter-session recognition accuracy of 27 gestures in CSL-HDEMG reached 31.3% (73.2% after per-trial voting) and 34.6% (81.4% after per-trial voting) with randomly selected 5 and 13 gestures for adaptation, respectively. The randomly selected 13 gestures are {11,27,10,5,18,7,1,25,19,12,20,26,22} and the 5 gestures are {11,27,10,5,18}.

### 3.3. Evaluation using CapgMyo

In this experiment, we evaluated inter-subject recognition of 8 gestures using the second recording session of DB-b and the recognition of 12 gestures using DB-c. We performed a leave-one-out cross-validation, in which each of the 10 subjects was used in turn as the test subject and a classifier was trained using the data of the remaining 9 subjects.

Our adaptation scheme enhanced inter-subject recognition with an per-frame improvement of 3.7% for DB-b and 2.3% for DB-c (shown in Table 4). For DB-b, the recognition accuracy reached 35.1% using only a single frame of HD-sEMG signals, and it reached 55.3% using simple majority voting over 150 frames. For DB-c, the recognition accuracy reached 21.2% for a single frame of HD-sEMG signals and 35.1% using simple majority voting over 150 frames.

We evaluated inter-session recognition for DB-b, in which the model was trained using data recorded from the first session and evaluated using data recorded from the second session. Our adaptation scheme enhanced inter-session recognition with an per-frame improvement of 6.2% (shown in Table 4). The recognition accuracy reached 41.2% for a single frame of HD-sEMG signals and 63.3% using simple majority voting over 150 frames.

Lastly, we evaluated classical AdaBN and our multi-stream AdaBN on both CSL-HDEMG and CapgMyo. As shown in Table 7, the resulting inter-session recognition accuracies are improved than classical AdaBN, especially when a large number of recording sessions are presented in the training set.

### 3.4. Evaluation using NinaPro

In this experiment, we followed the same inter-subject evaluation procedure as in a previous study [16]. We performed a leave-one-out cross-validation, in which each of the 27 subjects was used in turn as the test subject and a classifier was trained using the data of the remaining 26 subjects and calibrated using a small labeled subset of the test session. In each turn, we used the trials numbered 1, 3, 4, 5 and 9 of all 27 subjects for training and adaptation and used the trials numbered 2, 6, 7, 8 and 10 for testing. The training and calibration set were downsampled by a factor of 16, as in the previous study [16]. The model was first trained using the data from 26 subjects in the training set, then adapted using multi-stream AdaBN using the calibration set without label. Calibration with labeled data is an optional step of our framework. We also fine-tuned the adapted model using the labeled calibration set for a fair comparison. The recognition accuracies were 56.5% for a single frame of sEMG signals and 67.4% with simple majority voting over 40 frames, with a 100 Hz sampling rate. Patricia et al. [16] achieved a recognition accuracy of approximately 40% using an analysis window of 40 frames. These results demonstrate that our approach outperforms the state-of-the-art adaptation methods for gesture recognition, even when using sEMG signals from sparse multiple channels.

## 4. Discussion

In this work, we provide a new benchmark database—CapgMyo—for HD-sEMG-based gesture recognition. The CapgMyo database contains HD-sEMG data for 128 channels acquired from 23 intact subjects by using our newly developed acquisition device. We verified the technical quality of CapgMyo by showing that state-of-the-art classifiers could achieve higher recognition accuracy using HD-sEMG signals of CapgMyo than that using conventional sparse multi-channel sEMG.

We proposed a deep domain adaptation scheme for inter-session sEMG-based gesture recognition. Our multi-stream extension of AdaBN [55] improves inter-session accuracy, especially when when a large number of recording sessions are presented in the training set. We achieved state-of-the-art performance for inter-session and inter-subject scenarios, The recognition accuracy reached 85.0% on the CSL-HDEMG dataset using simple majority voting–a level approximately 9.6% higher than the latest work [2]. We showed that our adaptation scheme also performs well on conventional sparse multi-channel sEMG. The accuracy reached 67.4% when recognizing 52 finger gestures from NinaPro and using simple majority voting–a level approximately 27.4% higher than the state-of-the-art results [16]. Moreover, our adaptation scheme also works with a small amount of adaptation data and works if some categories are not performed yet during the process of interaction.

## 5. Conclusion and Further Works

Unlike typical inter-session gesture recognition requiring an explicit calibration for retraining or model-transferring, our unsupervised (no labels of data) adaptation works on data domain (no fine-tuning on the learned model), incrementally making statistics of all run-time data/samples closely similar to the one for training. It goes through the entire interaction process, not at a specific stage. Thus our work provides a foundation for developing more fluid and more natural muscle-computer interfaces. In future work, we plan to extend our framework using temporal models such as recurrent neural networks and accommodate dynamic transitional motions, in addition to static gestures.

The codes are available at https://github.com/Answeror/adamyo.

## Figures and Tables

**Table:** (**a**) Gestures in DB-a and DB-b (equivalent to Nos. 13–20 in NinaPro [48])

label	Description	Instance	label	Description	Instance
1	Thumb up	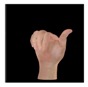	5	Abduction of all fingers	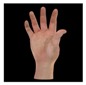
2	Extension of index and middle, flexion of the others	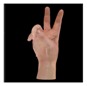	6	Fingers flexed together in fist	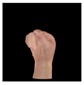
3	Flexion of ring and little finger, extension of the others	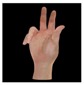	7	Pointing index	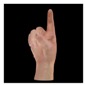
4	Thumb opposing base of little finger	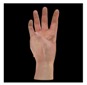	5	Adduction of extended fingers	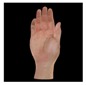

**Table:** (**b**) Gestures in DB-c (equivalent to Nos. 1–12 in NinaPro [48])

label	Description	Instance	label	Description	Instance
1	Index flexion	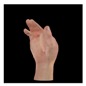	7	Little finger flexion	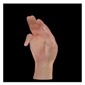
2	Index extension	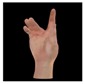	8	Little finger extension	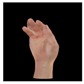
3	Middle flexion	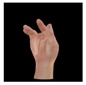	9	Thumb adduction	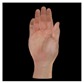
4	Middle extension	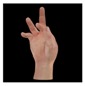	10	Thumb abduction	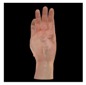
5	Ring flexion	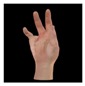	11	Thumb flexion	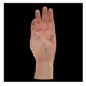
6	Ring extension	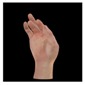	12	Thumb extension	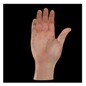

**Table:** (**c**) Max-force gestures (equivalent to gestures No. 5 and No. 6 in NinaPro [48])

label	Description	Instance	label	Description	Instance
100	Abduction of all fingers	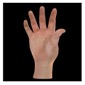	102	Fingers flexed together in fist	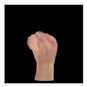

**Table:** sss_ggg.mat

Name	Type	Description
data	n×128 matrix	SEMG signals, where *n* is the number of frames.
gesture	n×1 matrix	The gesture ID of each frame, where 0 denotes the rest posture.
subject	Scalar	The subject ID.

**Table:** sss_ggg_ttt.mat

Name	Type	Description
data	1000×128 matrix	SEMG signals.
gesture	Scalar	The gesture ID.
subject	Scalar	The subject ID.
trial	Scalar	The trial ID.

**Table 3:** Subject ID in each sub-database. Some subjects participated in the acquisition of more than one sub-database. Each subject in DB-b took part in two recording sessions, which are marked with different IDs.

Subject ID	ID in DB-a	ID in DB-b		ID in DB-c
		Session 1	Session 2	
1	-	-	-	1
2	1	1	2	2
3	2	3	4	-
4	-	5	6	3
5	3	-	-	-
6	4	7	8	4
7	5	9	10	-
8	-	11	12	-
9	6	13	14	-
10	7	-	-	-
11	8	-	-	-
12	-	15	16	5
13	9	-	-	6
14	-	-	-	7
15	10	-	-	-
16	11	-	-	8
17	12	17	18	-
18	13	-	-	-
19	14	-	-	-
20	15	-	-	-
21	16	-	-	9
22	17	-	-	-
23	18	19	20	10

**Table 4:** Recognition accuracies (%) of different evaluation protocols on CSL-HDEMG (27 gestures) and CapgMyo (8 gestures for DB-b and 12 gestures for DB-c). The numbers are majority voted results using entire trial for CSL-HDEMG and 150 ms window (i.e., 150 frames) for CapgMyo, respectively. Per-frame accuracies are shown in parentheses.

	CSL-HDEMG	CapgMyo DB-b	CapgMyo DB-c
Intra-session	96.8 (55.8)	98.6 (85.6)	99.2 (84.6)
Inter-session without adaptation	62.7 (29.3)	47.9 (35.0)	
**Inter-session with adaptation**	**82.3 (35.4)**	**63.3 (41.2)**	
Inter-subject without adaptation		39.0 (31.4)	26.3 (18.9)
**Inter-subject with adaptation**		**55.3 (35.1)**	**35.1 (21.2)**

**Table 5:** Recognition accuracies (%) of the intra-subject evaluation with majority voting over each trial for CSL-HDEMG. Each row corresponds to one subject; the columns, denoted as S1 to S5, correspond to 5 sessions. The values in the second line of each row are those given by Amma et al. [2].

	S1	S2	S3	S4	S5	Avg	Std
A	**93.7**	**97.8**	**98.1**	**98.9**	**99.6**	**97.6**	**2.3**
	85.2	90.7	95.2	94.8	90.7	91.3	4.0
B	**96.3**	**97.0**	**97.0**	**95.9**	**97.0**	**96.8**	**0.5**
	83.7	92.2	94.1	88.9	90.0	89.8	3.9
C	**97.8**	**97.0**	**96.3**	**94.8**	**91.5**	**95.5**	**2.5**
	88.9	93.3	92.6	92.2	88.9	91.2	2.1
D	**94.8**	**98.5**	**99.3**	**89.9**	**98.5**	**96.2**	**3.9**
	87.8	92.2	87.0	85.4	84.8	87.4	2.9
E	**97.0**	**98.5**	**99.3**	**98.1**	**94.8**	**97.6**	**1.7**
	91.5	89.6	96.3	94.1	90.4	92.4	2.8
						**96.8**	**2.3**
						90.4	3.2

**Table 6:** Recognition accuracies (%) of the inter-subject evaluation with majority voting over each trial for CSL-HDEMG. Each row corresponds to one method; the columns, denoted by A to E, correspond to 5 subjects.

	A	B	C	D	E	Avg
[2]	60.6	73.0	62.3	53.3	45.3	58.9
[2] with calibration	83.9	77.2	71.0	66.5	78.4	75.4
Ours	69.9	71.3	61.2	59.4	51.8	62.7
Ours with adaptation	**87.7**	**87.9**	**72.1**	**80.0**	**83.6**	**82.3**

**Table 7:** Recognition accuracies (%) of inter-session evaluation with different adaptation methods. The numbers are majority voted results using entire trial for CSL-HDEMG and 150 ms window (i.e., 150 frames) for CapgMyo, respectively. Per-frame accuracies are shown in parentheses.

DB	CSL	DB-b		DB-c
		Inter-Subject	Inter-Session	
Training sessions	4	9	1	9
No AdaBN	62.5 (29.3)	39.0 (31.4)	47.9 (35.0)	26.3 (18.9)
Classical AdaBN	82.0 (35.2)	54.1 (33.8)	63.0 (41.1)	34.9 (20.9)
Multi-stream AdaBN	**82.3 (35.4)**	**55.3 (35.1)**	**63.3 (41.2)**	**35.1 (21.2)**

## Abbreviations

The following abbreviations are used in this manuscript:

MCI	muscle-computer interface
sEMG	surface electromyography
HD-sEMG	high-density surface electromyography
ConvNet	deep convolutional network
GMM	Gaussian mixture model
HMM	hidden Markov model
CCA	canonical correlation analysis
DAN	deep adaptation network
MK-MMD	multiple kernel variant of maximum mean discrepancies
GRL	gradient reverse layer
MVC	maximal voluntary contraction
BatchNorm	batch normalization
AdaBN	adaptive batch normalization
SGD	stochastic gradient descent
KNN	k-nearest neighbours
SVM	support vector machine
LDA	linear discriminant analysis
